# The Role of State Health Departments in Supporting Community-based Obesity Prevention

**Published:** 2011-06-15

**Authors:** Jamie M. Cousins, Sarah M. Langer, Cathy Thomas, Lori K. Rhew

**Affiliations:** Chronic Disease and Injury Section, North Carolina Division of Public Health; North Carolina State University, Raleigh, North Carolina. At the time of this study, Sarah Langer was affiliated with the University of North Carolina at Chapel Hill, Chapel Hill, North Carolina; during the writing of this article, she was affiliated with the North Carolina Division of Public Health, Raleigh, North Carolina; Eat Smart, Move More North Carolina, Raleigh, North Carolina; Eat Smart, Move More North Carolina, Raleigh, North Carolina. At the time of this study, Lori Rhew was affiliated with the North Carolina Division of Public Health, Raleigh, North Carolina

## Abstract

**Background:**

Recent national attention to obesity prevention has highlighted the importance of community-based initiatives. State health departments are in a unique position to offer resources and support for local obesity prevention efforts.

**Community Context:**

In North Carolina, one-third of children are overweight or obese. North Carolina's Division of Public Health supports community-based obesity prevention by awarding annual grants to local health departments, providing ongoing training and technical assistance, and engaging state-level partners and resources to support local efforts.

**Methods:**

The North Carolina Division of Public Health administered grants to 5 counties to implement the Childhood Obesity Prevention Demonstration Project; counties simultaneously carried out interventions in the community, health care organizations, worksites, schools, child care centers, and faith communities.

**Outcome:**

The North Carolina Division of Public Health worked with 5 local health departments to implement community-wide policy and environmental changes that support healthful eating and physical activity. The state health department supported this effort by working with state partners to provide technical assistance, additional funding, and evaluation.

**Interpretation:**

State health departments are well positioned to coordinate technical assistance and leverage additional support to increase the strength of community-based obesity prevention efforts.

## Background

More than two-thirds of North Carolina's adults and one-third of the state's children are overweight or obese (1,2). To reverse the growing obesity epidemic, the North Carolina Division of Public Health (NCDPH) supports community-based obesity prevention efforts through funding, training, and technical assistance. Strong partnerships at the state and local levels are necessary for these efforts. Community partners include community coalitions, recreation centers, religious organizations, physician's offices, child care providers, and schools.

Recent federal initiatives provide support for community-based interventions. In 2009, the Centers for Disease Control and Prevention (CDC) released the *Recommended Community Strategies and Measurements to Prevent Obesity in the United States* (3). In 2010, CDC granted federal stimulus funding to states, territories, tribal entities, and community initiatives for evidence-based obesity prevention strategies through the Communities Putting Prevention to Work initiative. For the community initiatives, CDC directly funded health districts serving more than 500,000 people; districts serving less than 500,000 could only apply through their state health department. Additionally, First Lady Michelle Obama launched the Let's Move campaign in 2010, calling for comprehensive, collaborative, and community-oriented solutions to the childhood obesity epidemic (4). These national initiatives promote community-based projects that address obesity in several settings and facilitate change on the personal, interpersonal, organizational, policy, and environmental levels (3,4). Shape Up Somerville: Eat Smart, Play Hard exemplified this multilevel, multisetting, community-based approach in Massachusetts (5) and was the inspiration for the Childhood Obesity Prevention Demonstration Project (COPDP).

As communities across the nation strive to reduce and prevent childhood obesity, state health departments can offer vital resources to enhance their efforts. In 2008-2009, the North Carolina General Assembly funded an innovative community-based project to reduce and prevent childhood obesity. Administered by NCDPH, COPDP offers valuable insight for state health departments supporting multilevel, multisetting, community-based obesity prevention. COPDP, which was supported with substantial state funding and state health department resources, resulted in positive change in 5 North Carolina counties. We discuss the role of NCDPH in this community-based childhood obesity prevention project. The purpose of this case study is to examine the role that state health departments play in supporting community-based efforts.

## Community Context

In 2010, North Carolina ranked 11th in the nation for childhood obesity among children aged 10 to 17 years (6). According to the 2007 National Survey of Children, 18.6% of North Carolina youth aged 10 to 17 years were obese (7), compared with 16.4% nationally (8). According to North Carolina's Child Health Assessment and Monitoring Program, one-third of children in North Carolina aged 10 to 17 years were overweight or obese in 2009 (2).

### NCDPH support for community-based obesity prevention

NCDPH dedicates resources to build the capacity of local health departments and local partnerships to address various health issues. Specific to healthful eating and physical activity, the NCDPH Physical Activity and Nutrition (PAN) Branch uses federal Preventive Health and Health Services Block Grant funds for the Statewide Health Promotion Program. This program supports policy and environmental change in 98 of North Carolina's 100 counties by building local capacity through funding, training, and technical assistance. In addition, local health departments, in collaboration with their partners, compete for grants of up to $20,000 annually to encourage physical activity and healthful eating in their communities by changing policies and environments.

The PAN Branch also uses CDC grant funding to cultivate and sustain state-level partnerships. Strong communication among state-level partners creates a more supportive statewide context for community-based initiatives. The most notable partnership is the Eat Smart, Move More North Carolina (ESMM-NC) leadership team, a multidisciplinary group of more than 60 statewide partner organizations. The PAN Branch provides staff support to the ESMM-NC leadership team, which guides the ESMM-NC movement (9) to increase opportunities for healthful eating and physical activity wherever people live, learn, earn, play, and pray.

In 2008, the PAN Branch, with support from North Carolina's state health director and chronic disease director, worked with ESMM-NC partners to advocate for state-supported, community-based projects to address childhood obesity. Partners used data such as the correlation between physical inactivity and academic performance and obesity-related health care costs to make a case for state funding to explore best practices in preventing childhood obesity. These partners created the COPDP plan based on the socioecological model, a multilevel, multisetting approach similar to the Shape Up Somerville project.

### Funding

In state fiscal year 2008-2009, the North Carolina General Assembly awarded $1.9 million to NCDPH for COPDP. The funding was originally in the budget as recurring but was ultimately designated as nonrecurring. The North Carolina General Assembly directed NCDPH to allocate the entire $1.9 million directly to local health departments to implement COPDP; however, the North Carolina General Assembly did not allocate funding for the state to provide administration and technical assistance. Consequently, NCDPH identified other resources to fund a state coordinator for the project and an external evaluation.

### Objective of COPDP

The objective of COPDP was to implement a set of multilevel, multisetting interventions for preventing and reducing obesity among children in a community. For the demonstration project, the state's objective was to learn lessons in community-based obesity prevention, how to support obesity-prevention efforts, and how to apply lessons learned in counties across the state.

## Methods

### The demonstration project framework

COPDP included 8 required and 4 optional community interventions (Table 1). Before the implementation of COPDP, each of the interventions had been implemented in some North Carolina communities, but no community had implemented all of them. The interventions targeted children and their adult role models in 6 settings: the community at-large, health care organizations, worksites, schools, child care centers, and religious organizations. COPDP incorporated 4 recommended strategies for physical activity, nutrition, and obesity prevention from the *Guide to Community Preventive Services*: 1) community-wide campaigns (10), 2) community-scale urban design and land-use policies (11), 3) worksite programs combining nutrition and physical activity (12), and 4) enhanced physical education classes in schools (13).

COPDP required simultaneous implementation of the interventions in 5 selected counties. A community-wide media campaign united each of the separate COPDP interventions under a single brand and ensured consistent obesity prevention messages. The counties used existing resources from the ESMM-NC statewide movement to raise awareness of the interventions and create a supportive environment for physical activity and healthful eating.

### County selection

NCDPH used a 2-step, competitive application process to select counties to implement COPDP, beginning with a request for applications in July 2008. Twenty-nine counties submitted brief proposals describing their local partnerships, experiences collaborating on community-based projects, and plans to coordinate COPDP. Of these, 11 were invited to submit full applications with a detailed plan for implementing each of the interventions. The applications also included descriptions of the capacity of each county's project coordinator and key staff. NCDPH awarded grants of $380,000 to each of 5 selected counties beginning on October 1, 2008. In accordance with the state fiscal year, the grant period ended May 31, 2009, giving the counties 8 months to implement the program. The counties ranged in population from 34,296 to 172,223 (14) and were geographically distributed across the state.

### State administration and technical assistance

NCDPH administered and provided technical assistance for COPDP. Preventive Health and Health Services Block Grant funds supported a full-time state coordinator at NCDPH. The state coordinator facilitated the involvement of state-level partners, developed a system for providing technical assistance, maintained constant contact with the county coordinators, and fostered sharing among the counties. These efforts ensured the efficient engagement of state-level expertise and resources, ongoing quality improvement, and problem solving to support the counties throughout COPDP. The state coordinator also monitored the counties' progress through site visits, monthly telephone calls, and reviews of written monthly summary reports.

Statewide partners assisted with COPDP by providing additional funding, technical assistance, and training (Table 2). For example, the North Carolina State Board of Education provided an additional $250,000 to school districts in the 5 counties to further support obesity prevention through coordinated school health programs as part of COPDP. In addition, COPDP counties were the first counties to participate in a new initiative to enhance physical education in schools across the state. Through this initiative, counties received technical assistance and training in an evidence-based physical education curriculum and in fitness-testing software. NCDPH developed a centralized technical assistance infrastructure (Figure) to support the counties with the COPDP interventions and to streamline this support as much as possible.

**Figure.**
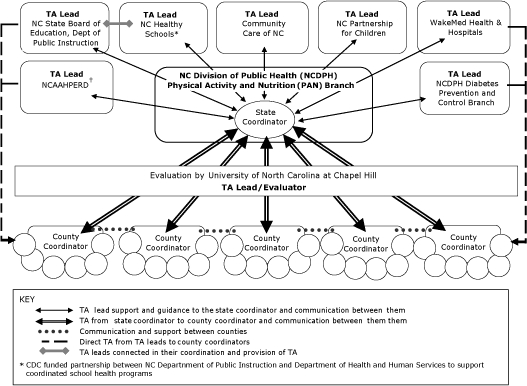
Technical Assistance (TA) Workflow for the Childhood Obesity Prevention Demonstration Project (COPDP). Abbreviations: NC, North Carolina; NCAAHPERD, North Carolina Alliance for Athletics, Health, Physical Education, Recreation, and Dance; CDC, Centers for Disease Control and Prevention.

**Table:** 

The COPDP state coordinator within the North Carolina Division of Public Health (NCDPH) collaborated with the partners shown to coordinate technical assistance to the COPDP communities: NCAAHPERD; the North Carolina State Board of Education, Department of Public Instruction; North Carolina Healthy Schools; Community Care of North Carolina; North Carolina Partnership for Children; WakeMed Health and Hospitals; and NCDPH Diabetes Prevention and Control Branch. These partners provided funding, technical assistance, and support for COPDP, as shown in Table 2. The North Carolina State Board of Education, Department of Public Instruction, and North Carolina Healthy Schools work together to support coordinated school health. CDC funds NC Healthy Schools, which is a partnership between the North Carolina Department of Public Instruction and the NC Department of Health and Human Services.
County coordinators received technical assistance from the state coordinator and for specific interventions, directly from NCAAHPERD, the North Carolina State Board of Education, Department of Public Health Instruction, WakeMed Health and Hospitals, and the NCDPH Diabetes Prevention and Control Branch. The 5 county coordinators from the health departments communicated with and supported each other. The evaluator from the University of North Carolina at Chapel Hill worked with the state coordinator and county coordinators on the evaluation plan, data collection, and the evaluation report.

### County implementation

COPDP grants went to local health departments in each county, which collaborated with county partnerships to implement the project. County-level implementation was directed by a county coordinator who worked closely with the state coordinator to ensure the fulfillment of all grant requirements.

The county partnerships were well established before COPDP. Once funded, each partnership assessed its membership and added members as needed to include representation from all intervention settings. The number of agencies represented in each partnership ranged from 9 to 16. The partnerships garnered resources (eg, volunteer time, space for classes, access to organizations and individuals, support from local leaders and boards) through their existing connections and relationships.

### Evaluation

NCDPH funded the evaluation of COPDP through the Prevention Research Center at the University of North Carolina-Chapel Hill. A full-time COPDP evaluator worked with the state and county coordinators to 1) collect output data describing implementation, 2) measure intermediate outcomes describing changes in the communities, 3) examine the role of county partnerships in facilitating the success of the project, and 4) identify potential long-term outcome measures. The COPDP evaluator also conducted interviews and focus groups, provided data analyses, and summarized findings. NCDPH engaged statewide partners in discussion of the evaluation results to share successes and opportunities for further collaboration. The institutional review board of the University of North Carolina at Chapel Hill approved the evaluation.

## Outcome

### Changes

The existence of COPDP resulted in immediate policy and environmental changes in the community, health care organizations, worksites, schools, child care centers, and religious organizations (Table 3). In the 5 counties, 42 child care providers made changes that affected more than 2,400 children. Policy changes to support physical activity and healthful eating in hospital and school worksites affected more than 13,800 employees. Sixty-six children at risk for type 2 diabetes completed a 36-session prevention program; of the 25 children who began the program with high triglycerides, 22 reduced their triglycerides and 14 achieved a normal range. Additionally, more than 6 miles of greenways and sidewalks were constructed or designed. In some cases, the effect of these changes reached beyond county and even state lines. For instance, a child care center's request for skim and 1% milk led to new food purchasing and distribution policies, which provided more healthful milk options for affiliated child care centers across the nation.

The media campaign blanketed communities with consistent messages that tied interventions together. One woman claimed that seeing and hearing the same ESMM-NC messages in multiple places — both in the community and in her workplace — made her feel connected to a larger effort. NCDPH and the counties documented success stories like these to illustrate the personal effect on community members. These stories were used to educate policy makers and stakeholders.

### Lessons learned

The short time line, high visibility, and the large scale of COPDP presented challenges. Once the funding was awarded, NCDPH quickly leveraged support from state partners for the 5 counties in the form of additional funding, training, and technical assistance for COPDP. NCDPH's long-standing relationships and collaboration with state partners made this possible.

The 8-month time line for the COPDP presented several challenges. Incorporating grant activities, such as staff training and a new physical education curriculum for kindergarten through 8th grade, was challenging for schools because lesson plans and teacher professional development days had already been set for the year. Seasonal effects also limited progress; for example, farmers' markets closed for winter just as COPDP began.

NCDPH worked with counties to balance the need for fidelity in implementation with the need for flexibility to adapt interventions to the local context. County health departments were asked to adhere to specific grant requirements, even if they adapted activities. In some cases, counties were simply not able to implement the interventions as specified because of time and other constraints. For example, several counties adapted the enrollment criteria for 1 intervention because they could not otherwise recruit enough participants in the given time frame.

A community-based health initiative's duration affects its sustainability, and several years of implementation are needed to institutionalize the desired change (15). Although COPDP was designed for 5 years of funding, only 1 year of funding was initially awarded. NCDPH and the counties knew that continued funding was tentative; when a state fiscal crisis ensued in 2009, funding was not allocated for COPDP. Counties developed sustainability plans, but without additional resources, not all of the interventions could be continued. Likewise, funding was not available to evaluate the outcomes of COPDP beyond the 8-month time frame. Further evaluation of this project is needed to track the counties' continued efforts and measure long-term effects. Additional results, lessons learned, and success stories are available on the ESMM-NC website (www.EatSmartMoveMoreNC.com/ObesityDemo/ObesityDemo.html).

## Interpretation

Communities are an essential forum for obesity prevention, and state health departments are uniquely positioned to support and enhance these efforts. States can leverage support and help to build the local capacity needed to implement comprehensive projects that effect change in multiple community settings. NCDPH found that many North Carolina counties have already united with partners for obesity prevention and are well positioned to increase the scale of their efforts. Examples of NCDPH's long-term commitment to strengthen the capacity of local partnerships and public health departments include the Statewide Health Promotion Program and the distribution of community grants.

State health departments must think strategically about investing in community-based health initiatives. States funding large-scale, multilevel, and multisetting obesity prevention projects should consider a community partnership's previous experience in collaborating on similar projects. Prior collaboration and existing relationships equip local partnerships to work through the challenges of these initiatives.

States should also consider the leadership capacity and skills of local coordinators. Focus groups and in-depth interviews with key informants in the COPDP counties revealed that skilled county coordinators were instrumental to the success of the effort. From the community perspective, the coordinators were the leaders of local partnerships that provided opportunities to network, share information, solve problems, and celebrate successes. From the state perspective, the county coordinators were necessary for troubleshooting, problem solving, engaging state technical assistance, and facilitating data collection.

Given the state budget process, funding often comes with short notice and duration. When offering grants for community-based projects, state health departments should allow themselves enough time to develop clear expectations and allow grantees enough time to plan effectively and secure partner commitments. More time before the start of COPDP would have allowed NCDPH and partners to better prepare materials, organize technical assistance, and develop data collection tools. Additionally, securing several years of funding and state resources to support COPDP would have enhanced the degree of sustainable change. To be effective, state health departments must be prepared to work within the context of short timelines and high expectations. As shown by COPDP, maintaining strong partnerships can lead to quick mobilization and additional resources when opportunities arise.

Finally, state health department staff time and resources are needed for community-based childhood obesity prevention programs. The state coordinator for COPDP provided guidance on implementing evidence-based and best practices, engaged state-level partners, and coordinated technical assistance. Addressing obesity is complex, requiring expertise in nutrition, physical activity, urban planning, sustainable food systems, school health, and other disciplines. Although some of this expertise exists among the state health department staff, much of it requires collaboration with external partners. State health department staff are well positioned to work with partners to coordinate technical assistance and leverage additional support to increase the strength of community-based obesity prevention efforts.

## Figures and Tables

**Table 1:** Childhood Obesity Prevention Demonstration Project (COPDP) Interventions, North Carolina, 2008-2009

Setting	Required or Optional	**Intervention**	**Description**
Community	Required	Partnership development	Assess partnership (eg, leadership's effectiveness, member satisfaction), offer member training, and engage members in strategic planning and sustainability planning
	Required	Built environment	Complete construction or design phases of an existing project in the county's master plan
	Required	Health communication and social marketing	Implement a community-wide campaign by using Eat Smart, Move More North Carolina messages and branding in conjunction with local partnership branding and marketing of other COPDP interventions
	Optional	Farmers' market/farm stands	Create or enhance farmers' markets or farm stands to improve access to fresh produce
Health care	Required	WakeMed ENERGIZE! Program	Establish clinical referral process and conduct an intensive 12-week program for children aged 10-18 years with or at risk for diabetes and other metabolic diseases
	Required	Pediatric obesity clinical tools and training	Provide resources and training for health care providers or practices in assessment and treatment of pediatric obesity
Worksite	Required	Hospital worksite wellness	Support hospital wellness committees to change policies and environments and offer initiatives to improve employee health
	Optional	School worksite wellness	Support school wellness committees to change policies and environments and offer programs to improve staff health
School	Required	NCAAHPERD's In-School Prevention of Obesity and Disease Program	Train kindergarten through high school physical education teachers on the SPARK curriculum and FITNESSGRAM assessments, including calculation of body mass index
	Optional	Other coordinated school health interventions	Improve health education, nutrition services, and healthful school environments
Child care center	Required	NAP SACC program	Train staff and improve policies, practices, and environments to support physical activity and healthful eating
Faith-based organization	Optional	Faith community intervention	Support leadership and wellness committees in changing policies and environments and offering programs to improve member health

Abbreviations: NCAAHPERD, North Carolina Alliance for Athletics, Health, Physical Education, Recreation, and Dance; SPARK, Sports, Play, and Active Recreation for Kids; NAP SACC, Nutrition and Physical Activity Self-Assessment for Child Care.

**Table 2:** Role of State Partners in Implementing the Childhood Obesity Prevention Demonstration Project (COPDP)

**Agency**	**Role**	**Activities**
North Carolina Division of Public Health (NCDPH), Physical Activity and Nutrition Branch	Funding, administration, technical assistance	Provided funding for a full-time state coordinator and external evaluation of COPDP. Administered grants, coordinated technical assistance from NCDPH and partners, and supported evaluation of the project
NCDPH — North Carolina Office of Healthy Carolinians and Health Education	Advocacy support	Supported local partnerships in implementing the COPDP and highlighted the project at the annual Healthy Carolinians conference
NCDPH — North Carolina Diabetes Prevention and Control Branch	Technical assistance	Provided technical assistance in implementing the WakeMed ENERGIZE! Program
NCDPH — North Carolina State Center for Health Statistics	Technical assistance	Provided technical assistance on developing and implementing survey tools for evaluation of COPDP
North Carolina State Board of Education	Funding, technical assistance	Provided funding to school districts (total $250,000) in COPDP counties to support coordinated school health interventions as part of COPDP; provided technical assistance on these interventions
North Carolina Healthy Schools^a^	Technical assistance	Provided technical assistance on COPDP interventions to support coordinated school health programs
WakeMed Health and Hospitals	Training, technical assistance	Provided training to health care practitioners in screening program participants and provided training and technical assistance to local health departments and partners in implementing the WakeMed ENERGIZE! Program
North Carolina Alliance for Athletics, Health, Physical Education, Recreation, and Dance	Training, technical assistance	Coordinated SPARK curriculum training for physical education teachers; provided technical assistance in implementing the In-School Prevention of Obesity and Disease program
Community Care of North Carolina	Consultation	Consulted with NCDPH and COPDP counties on implementing an intervention to train health care practitioners on the use of pediatric obesity clinical tools
North Carolina Partnership for Children	Consultation, technical assistance	Consulted with NCDPH and COPDP counties on implementing the NAP SACC program; provided technical assistance to local Partnership for Children agencies as requested
University of North Carolina at Chapel Hill's Prevention Research Center	Evaluation, technical assistance	Developed and implemented an evaluation plan for COPDP; provided technical assistance to the counties and NCDPH in data collection and analysis

Abbreviations: SPARK, Sports, Play, and Active Recreation for Kids; NAP SACC, Nutrition and Physical Activity Self-Assessment for Child Care.

a CDC-funded partnership between the North Carolina Department of Public Instruction and North Carolina Department of Health and Human Services to support coordinated school health programs.

**Table 3:** Childhood Obesity Prevention Demonstration Project (COPDP) Evaluation Highlights, North Carolina, 2008-2009

**Setting**	**Intervention**	**Evaluation Highlights**
Community	Partnership development	Five county partnerships completed a pre- and post-partnership self-assessment tool and conducted at least 2 trainings for partnership members.
	Built environment	Four counties built a total of 4.14 miles of sidewalks and greenways directly accessible to more than 7,300 residents in adjacent neighborhoods; 1 county completed the design and engineering phase for 1.5 miles of greenway.
	Health communication and social marketing	5.7% more residents were familiar with the ESMM-NC campaign at the end of the project.
	Farmers' market/farm stands	Four counties completed action plans with initiatives to increase access to 14 farmers' markets or farm stands.
Health care	WakeMed ENERGIZE! program	Children (n = 66) completed at least 30 of 36 sessions over a 12-week period.
	Pediatric obesity clinical tools and training	Clinicians (n = 133) were trained on the importance and use of the pediatric obesity tools.
Worksite	Hospital worksite wellness	Six hospital systems implemented policies, environmental changes, and initiatives with the potential to affect 13,800 employees.
	School worksite wellness	Four school worksite wellness committees in 3 counties implemented policies, environmental changes, and initiatives with the potential to affect more than 2,700 staff.
School	NCAAHPERD's In-School Prevention of Obesity and Disease program	More than 180 teachers were trained in the SPARK curriculum.
	Other coordinated school health interventions	Five counties implemented initiatives, including a pilot farm-to-school program, menu labeling, new vending policies, and installation of steamers in school cafeterias.
Child care center	NAP SACC program	Forty-two child care centers implemented a total of 266 policy and environmental changes to support healthful eating and physical activity.
Faith-based organization	Faith community intervention	Nine faith communities implemented policies, environmental changes, and initiatives reaching an estimated 758 members.

Abbreviations: ESMM-NC, Eat Smart, Move More North Carolina; NCAAHPERD, North Carolina Alliance for Athletics, Health, Physical Education, Recreation, and Dance; SPARK, Sports, Play, and Active Recreation for Kids; NAP SACC, Nutrition and Physical Activity Self-Assessment for Child Care.
